# Vimentin and p53 Immunoreactivity in Cases of Traumatic Brain Injury

**DOI:** 10.3390/jpm15040135

**Published:** 2025-03-31

**Authors:** Alice Chiara Manetti, Alessandra De Matteis, Gabriele Napoletano, Raffaele La Russa, Aniello Maiese, Paola Frati

**Affiliations:** 1 Department of Public Health and Infectious Diseases, Sapienza University of Rome, 00161 Rome, Italy; alicechiara.manetti@uniroma1.it; 2 Department of Anatomical, Histological, Forensic and Orthopedic Sciences, Sapienza University of Rome, 00161 Rome, Italy; alessandra.dematteis@uniroma1.it (A.D.M.); gabriele.napoletano@uniroma1.it (G.N.); paola.frati@uniroma1.it (P.F.); 3 Department of Clinical Medicine, Public Health, Life Sciences, Environmental Sciences, University of L’Aquila, 67100 L’Aquila, Italy; raffele.larussa@univaq.it

**Keywords:** traumatic brain injury, forensic neuropathology, vimentin, p53, vitality

## Abstract

**Background**: Traumatic brain injury (TBI) is one of the main causes of death in trauma pathology, especially among the youngest victims. After having evaluated the causality relationship between damage to the brain tissue and death, pathologists should try to estimate the duration between the TBI and death. Immunohistochemistry could be used in this field as a personalized medico-legal approach. This study aims to evaluate the possible role of vimentin and p53 as TBI markers to assess vitality and date the TBI. **Methods**: Twelve cases of TBI deaths were selected (two women and ten men, with a mean age of 46.83 years). In seven cases, death occurred immediately after the trauma, while in the others, death occurred after some days. An immunohistological study of brain samples using anti-p53 and anti-vimentin antibodies was performed. A semi-quantitative scale was adopted to grade the immunohistochemical reaction. **Results**: Our results showed a strong relationship between the p53 immunoreaction grade and TBI (X-squared value 10.971, *p*-value < 0.01), suggesting that p53 expression is enhanced in TBI cases. Vimentin is more expressed when the PTI is longer. Vimentin-immunoreaction was weaker than p53-immunoreaction (+0.75 vs. +1.83 mean values, respectively) in a group predominantly including short post-traumatic interval cases. **Conclusions**: The present research is limited by the small sample size; however, the molecules tested, vimentin and p53, have shown great potential to be used, in addition to others, as biological markers for the diagnosis and timing of TBI.

## 1. Introduction

Traumatic brain injury (TBI) is one of the main causes of death in trauma pathology, especially among the youngest victims. The types of trauma that can provoke TBI vary from motor vehicle accidents to falls from heights, sports accidents, etc. [1]. TBI can be described as damage to the brain caused by different kinds of forces applied to the head [2]. Indeed, a lesion to the cerebral tissue could be derived from either a direct head trauma or an indirect projection of high kinetic forces applied to the body, as often happens in vehicle crashes [3]. Regardless of the cause, TBI is characterized by altered brain functions and/or anatomical alterations. Considering the forensic pathology perspective, TBI has always represented an issue. These cases can be very variable, and a specific approach may be necessary. The first question the pathologist should focus on is if the brain lesions are the cause of death. Once the causality relationship between the damage to the brain tissue and death has been examined, the second most important question concerns the duration between the TBI and death. Understanding whether the victim survived the injury, and how long, could be fundamental for the judicial interpretation of cases. The immediate effects of head trauma are called primary brain injuries (PBIs). They can be focal or diffuse, according to the localization of the damage. Even if there is not a unique relationship between the type of trauma and the type of brain damage, low-energy traumas usually cause focal lesions, while mild and severe head traumas cause both diffuse and focal lesions [4]. Examples of focal lesions are brain contusions, hemorrhages (intracerebral, subdural, or extradural), and necrotic areas due to reduced blood supply. Diffuse brain lesions like diffuse traumatic axonal injury (tDAI) and diffuse hypoxic/ischemic damage involve the whole brain tissue and are the consequence of non-contact forces [5,6]. In tDAI, axons are rapidly stretched, and this causes damage to their cytoskeleton, impairing axoplasmic transport. Using beta-APP immunohistochemistry antibody, beads, varicosities, and disconnected axons have been observed within 35 min after a severe head injury [7]. The swelling of the tissue that occurs soon after a stretch trauma further aggravates axonal damage.

Secondary brain injuries (SBIs) could be defined as the delayed progression of PBI. Several biochemical and molecular mechanisms of damage have been described to explain SBIs. Apoptosis plays a major role in SBI. Beer et al. demonstrated that p18, the active caspase-3 subunit, is increased in the neurons, astrocytes, and oligodendrocytes of injured brain tissue in rats from 6 to 72 h after trauma [8]. Accordingly, Franz et al. (2002) found that Bid cleavage is enhanced from 6 h to 7 days after TBI in rats [9]. Bid is a pro-apoptotic protein that belongs to the Bcl-2 family [10]. Larner et al., in 2005, investigated the expression of Caspase 7 after a TBI in a rat model [11]. They chose caspase 7 because it is usually not present in brain tissue. They used a semiquantitative real-time polymerase chain reaction (PCR) to evaluate the caspase 7 mRNA levels and found that they were enhanced in TBI. Different peak caspase 7 expression was obtained with different timing, depending on the cerebral area: in the traumatized cortex, the peak was on the fifth day after the trauma, while in the hippocampus, it was between 6 and 24 h. Several studies have been conducted to evaluate the role and timing of the production of apoptotic proteins after a TBI. However, most of them are based on animal models. In human TBI, Dressler et al. showed that the earliest positive caspase 3 reaction in cortical neurons was evident after a post-traumatic interval of 80 min [12]. Considering the previous knowledge in this field, our research group focused on the identification of molecules that could be used as markers of TBI for diagnostic and timing purposes [13]. This could be useful in judicial cases, it is fundamental to provide solid scientific evidence to corroborate the interpretation of the events. In our previous research, we demonstrated that the expression of FOXO3a, a pro-apoptotic transcription factor, is enhanced in TBI. Furthermore, the FOXO3a immunoreaction was progressively more intense with increased survival time [14]. The current study aims to evaluate the possible role of vimentin and p53 as TBI markers.

## 2. Materials and Methods

### 2.1. Study Group Selection

The autopsy databases of the Legal Medicine and Forensic Institutes of the “Sapienza” University of Roma were retrospectively reviewed to identify cases of TBI. The autopsy and investigation reports were analyzed. Inclusion criteria for the study group were as follows: the cause of death was related to TBI, the autopsy was performed within 36 h of the death, and the cause of death was clearly identified. Decomposed bodies or those with initial signs of putrefaction were excluded, as well as cases with a history of cerebral or vascular disease. Twelve cases of TBI deaths were selected. The resulting study group was composed of 2 women and 10 men, with a mean age of 46.83 years. In 7 cases, death occurred immediately after the trauma (PTI = 0), while in the other cases, death occurred some days after the traumatic event (PTI between 2 and 12 days: in 1 case, 2 days; in 2 cases, 4 days; in 1 case, 5 days; and in 1 case, 12 days). A summary of the main characteristics of the study group is provided in Table 1.

From the same database, 12 cases of non-traumatic death were also selected as the control group (five women and seven men, with a mean age of 44.5 years and median age of 48.5 years). No traumatic event preceding the death was described in these cases. Again, only cases in which the autopsy was performed within 36 h of death were included. The exclusion criteria were the same as the case group. For both groups, the cerebral tissue samples were collected during the autopsy and then immediately fixed in 10% buffered formalin to prevent any putrefactive phenomena. The samples were 3 cm^2^ in size. In the case group, the samples were obtained from the cerebral cortex of the traumatic lesions and near the hemorrhagic areas, while in the control group, the cortex was sampled in the standard areas, such as the frontal cortex, temporal cortex, and occipital cortex.

The processing of the data reported in this paper is covered by the general authorization to process personal data for scientific research purposes granted by the Italian Data Protection Authority (1 March 2012 as published in Italy’s Official Journal no. 72 dated 26 March 2012) since the data do not entail any significant personalized impact on data subjects. Our study did not involve the application of experimental protocols; therefore, it did not require approval by an institutional and/or licensing committee. The bodies included in this study were autopsied by order of the Italian Judicial Authority. In all cases, local prosecutors opened an investigation, ordering an autopsy to be performed to clarify the exact cause of death. Therefore, according to Italian law, no ethical approval was needed. However, the present study was conducted with respect for the deceased involved, and any data were anonymized to guarantee the privacy of each subject.

### 2.2. Sample Preparation and Microscopic Analysis

**Figure 1:**
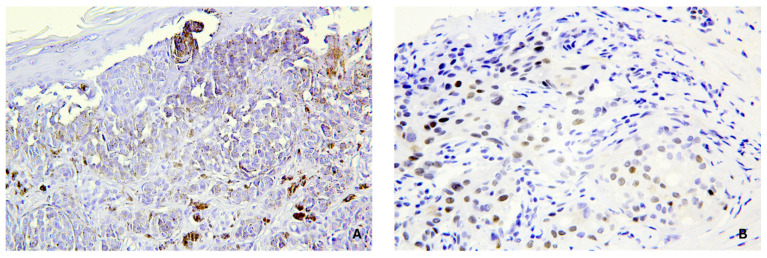
Positive controls. (**A**) Skin sample positive for vimentin; (**B**) breast cancer sample positive for p53.

Routine histological analysis with hematoxylin–eosin (H&E) staining was performed. In addition, an immunohistochemical investigation of cerebral samples was performed. The fixed samples were washed with phosphate-buffered saline (PBS), and subsequent dehydration was carried out using a graded alcohol series. After dehydration, samples were cleared in xylene and embedded in paraffin. Sections measuring 4 µm were mounted on slides and covered with 3-amminopropyltriethoxysilane (Fluka, Buchs, Switzerland). Antigen retrieval was carried out using an EDTA buffer in a pressure steamer at 100 °C for 90 min. Slides were stained on an automated immunostainer (Dako Cytomation, Glostrup, Denmark) using anti-vimentin and anti-p53 antibodies (Clone V9–ScyTek and DO-7 Leica, respectively). Before staining the study group’s samples, both types of antibodies were tested on positive controls. The positive controls are shown in Figure 1.

### 2.3. Semi-Quantitative Analysis

Twenty observations were made for the different slides of each immunohistochemical section (100-fold magnification). A confocal microscope was also used to perform a three-dimensional reconstruction (True Confocal Scanner, Leica TCS SPE, Cambridge, UK). The staining intensity was evaluated using a semi-quantitative scoring scale to grade the immunohistochemical reactivity (brownish staining). The scale varied from 0 to +3, comparing the intensity in the samples with the positive control, as shown in Table 2.

All measurements were carried out at the same image magnification (×10). The evaluations were carried out separately for each sample, using a double-blind method. In cases of divergent scoring, a third observer decided the final score.

### 2.4. Statistical Analysis

To compare the two variables (vimentin and p53 immunoreactivity) among the TBI and non-TBI groups, a Pearson’s Chi-squared test with Yates’ continuity correction was used. Then, the semi-quantitative results were reported in a Cartesian chart for each antibody: on the *y*-axis, the grade; on the *x*-axis, the PTI (hours). An analysis of the correlation between the two variables (immunoreaction and PTI) was performed using the Spearman rank correlation test.

## 3. Results

The results of the immunohistochemical analysis of the brain samples obtained from the 12 cases of TBI are reported in Table 3.

**Figure 2:**
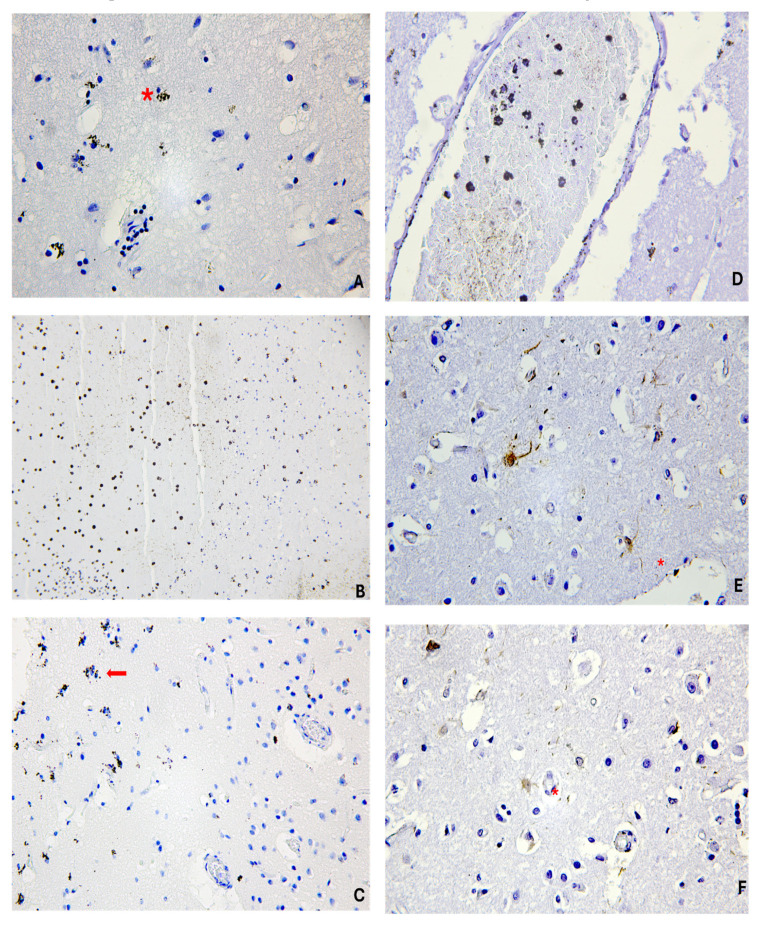
Vimentin and p53 positivity in TBI. (**A**) Monocyte/macrophage-like cells (red asterisk) with cytoplasm positive for p53 in pericontusional zone of cerebral cortex (p53, original magnification × 600). (**B**) Leukocytes positive for p53 in intracerebral hemorrhage (p53, original magnification × 100). (**C**) Glial cells (red arrow) and neurons positive for p53 in pericontusional zone of cerebral cortex (p53, original magnification × 400). (**D**) Leukocytes positive for vimentin within cerebral vessel (vimentin, original magnification × 100). (**E**) Astrocytes (red asterisks) positive for vimentin in pericontusional zone of cerebral cortex (vimentin, original magnification × 600 and × 400 (**F**).

As described, vimentin showed a positive reaction mainly in the cases with a PTI of greater than one day, while in the remaining cases (cases 2, 6, 7, 8, 9, 11, and 12), vimentin was negative or scarcely positive. On the other hand, p53 reactivity was positive in almost all cases (9 cases of 12). The three p53-negative cases had a PTI of shorter than one day. Some examples of the immunoreaction in our cases are shown in Figure 2.

The corresponding grade using the semi-quantitative scale is presented in Table 4.

Vimentin immunoreactivity was between 0 and +2; in no cases was the vimentin immunoreactivity +3. The mean value of vimentin immunoreactivity in the cases was +0.75, with a median of +0.5.

p53 showed an immunoreactivity between 0 and +3, with a mean value of +1.83 and a median of +2.0.

The results of the immunohistochemical analysis of the brain samples obtained from the 12 controls are reported in Table 5.

As shown, only in two cases was a positive reaction found: the brain samples of case 16 were slightly positive for p53, while the brain samples of case 24 were slightly positive for vimentin. The contingency table is shown in Table 6.

**Figure 3:**
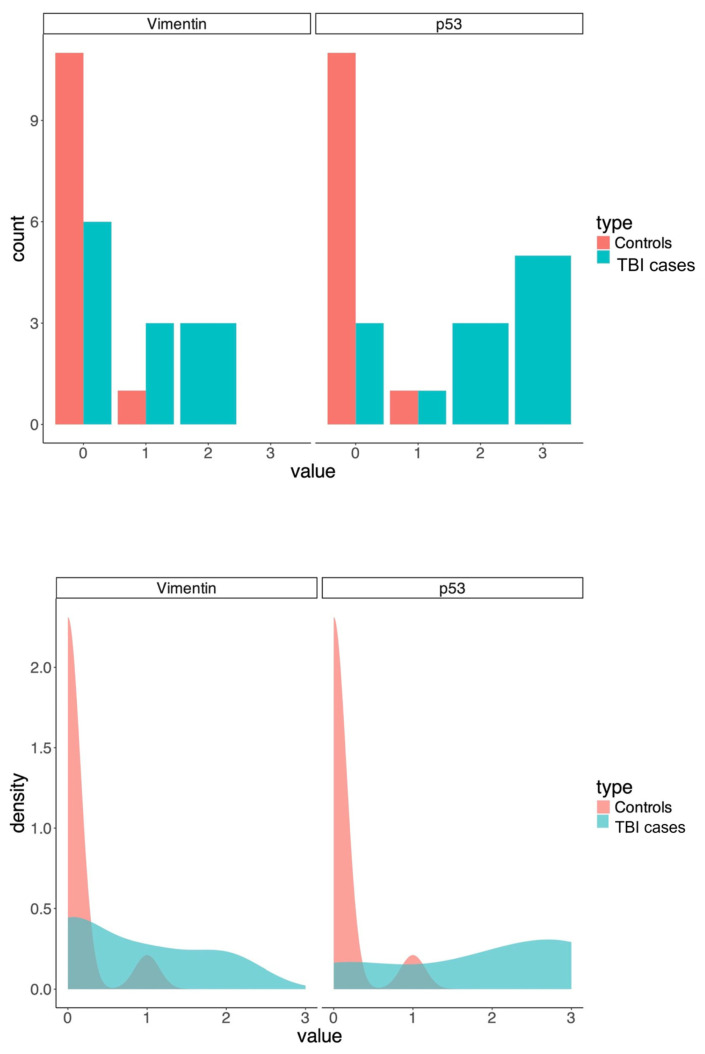
Graphs showing the different grade distribution among the two groups. The *y*-axis shows the number of cases, while the *x*-axis shows the immunoreaction grade.

Graphs representing the different grades of immunoreaction between the TBI group and the control group are shown in Figure 3.

Performing Pearson’s Chi-squared test with Yates’ continuity correction used on the contingency table (Table 6) produced the following results:

The X-squared value for the vimentin-immunoreaction comparison between the cases and controls is 5.042, df = 1, *p*-value = 0.02474 (<0.05). The hypothesis of a correlation between the two variables is supported.

The X-squared value for the p53-immunoreaction comparison between the cases and controls is 10.971, df = 1, *p*-value = 0.0009253 (<0.01). The hypothesis of a correlation between the two variables is supported.

The Spearman rank correlation analysis produced the following results: The rho value evaluating the correlation between the vimentin-immunoreaction and PTI is 0.9029, S = 27.759, *p*-value = 0.00005753 (<0.01). The hypothesis of a correlation between the two variables is supported.

The rho value evaluating the correlation between the p53-immunoreaction and PTI is 0.09873785, S = 257.76, *p*-value = 0.7601 (>0.05). The correlation between the two variables is very weak, and the analysis is not supported by a sufficient level of significance.

**Figure 4:**
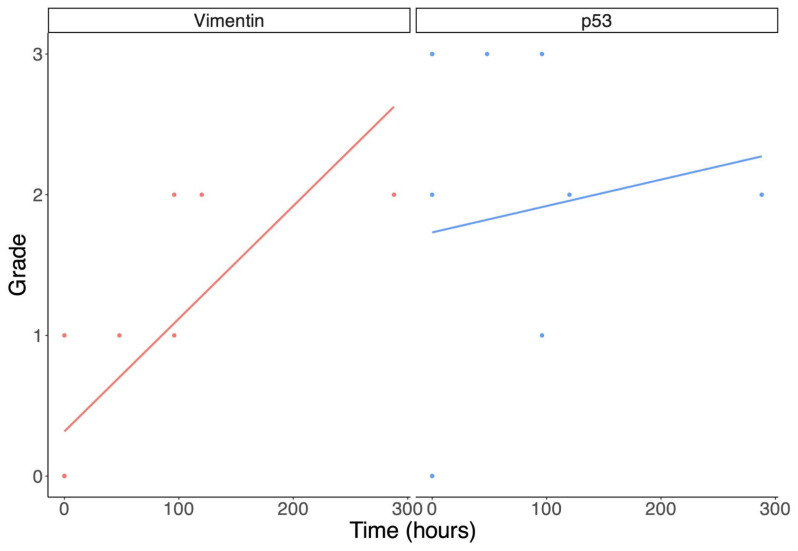
Spearman rank correlation between the immunoreaction grade and the time between trauma and death. Vimentin is on the left and p53 is on the right.

The results of the Spearman rank correlation analysis are shown in Figure 4.

These data highlight how this specific and personalized approach to diagnosing and dating TBI using anti-vimentin antibodies could be useful. However, it should be used in conjunction with other markers rather than alone. In the case of p53, the same observations cannot be made as the marker was diffusely expressed in all cases.

## 4. Discussion

Our study aimed to evaluate the relationship between the immunoreactivity of two markers, vimentin and p53, in cases of TBI. We selected suitable TBI cases from our autopsy database and 12 cases were obtained. The immunoreactivity of the two markers was tested on brain samples collected during the autopsy. Then, a semi-quantitative grading system was used to quantify the immunoreaction. The same process was performed with twelve control cases. Our results demonstrated a strong relationship between p53 and TBI (X-squared value 10.971, *p*-value < 0.01), suggesting that the p53-immunoreaction grade is higher in TBI cases. Therefore, our initial hypothesis that p53 antibodies may be used as a diagnostic marker of TBI in immunohistochemical analysis of brain samples is supported. p53 is an oncosuppressor protein involved in apoptosis regulation. Apoptosis is a type of organized cell death characterized by the need for energy to be initiated. p53 can bind the DNA, act as a transcription factor, and control various intracellular and intranuclear molecular pathways, such as genome surveillance and repair. In physiological conditions, p53 induces the death of cells that have undergone DNA damage [15]. Apoptosis plays a fundamental role in brain cell damage after a TBI [16]. The correlation between vimentin and TBI is slightly lower (X-squared value 5.042, *p*-value < 0.05). This result suggests that vimentin is not as good as a diagnostic marker of TBI compared to p53, even if the correlation between the vimentin-immunoreaction and TBI still demonstrates a good level of statistical significance. A possible explanation of these results may rely on the nature of the two chosen markers. As known, p53 is a pro-apoptotic protein [17]. This means that it is produced when some kind of cell damage occurs, such as DNA damage [18]. In TBI, the neurons suffer a mechanical injury that could induce the release of cell signals that promote programmed cell death. Indeed, it is believed that this process happens in the initial phases of brain injury [19]. Vimentin is a protein belonging to the family of intermediate filaments (IFs). It is mainly expressed in mesenchymal cells and is frequently used as a marker to study the development of cells and tissues [20]. The vimentin gene has been conserved through evolution among different species; therefore, it supposedly plays a key role in physiological cell mechanisms [21]. Experiments conducted on vimentin-deficient (vimentin −/−) mice cells have shown that there are consequent glial changes, an impaired healing process, fibroblast migration defects, and other dysfunctions [22,23,24]. In adult mice, vimentin seems to be expressed prevalently in muscle and central nervous system mesenchymal cells [25]. Vimentin also plays a role in cancer invasiveness. Indeed, the increased expression of vimentin correlates with the migration of neoplastic cells, metastasis, and poor prognosis [26]. When first identified, vimentin was thought to be an intracellular protein. However, an extracellular distribution has been demonstrated in the plasma membrane [27].

Vimentin has previously been suggested to be a marker of TBI. It has been studied with the aim of evaluating the presence of astrocyte changes [28]. O’Leary and colleagues demonstrated that vimentin immunoreactivity is generally weak in normal brain tissues, with a region-dependent distribution; some brain areas were completely negative [29]. In the forensic field, immunohistochemical antibodies against vimentin were used to date TBI by R. Hausmann and P. Betz. They showed that activated microglia/macrophages were positive for vimentin in the contused zone in cases with post-traumatic intervals ranging from 22 h to 6 days. Between 6 days and 4 weeks, the authors observed microglia-like cells and astrocytes with numerous fibrous processes in the perilesional zone, which could be indicative of a reparative response to the injury [30]. Vimentin seems to be produced when a harmful stimulus is provided over a longer duration. Indeed, vimentin expression has been demonstrated to be augmented in chronic brain disease [31]. Our TBI group was composed of 12 cases; of those, death occurred immediately after trauma in 7 cases (58.33%), while in the other 5 cases, death occurred some days after the trauma (in 1 case, 2 days; in 2 cases, 4 days; in 1 case, 5 days; and in 1 case, 12 days). Our hypothesis is that p53 is overexpressed in the first phase of brain injury, while vimentin is more expressed when the PTI is longer. This seems to be supported by our results: the vimentin-immunoreaction was weaker than the p53-immunoreaction (+0.75 vs. +1.83 mean values, respectively) in the group dominated by short-PTI cases. Indeed, vimentin-immunoreactivity was negative in almost all cases with PTI = 0. Upon evaluating the scientific literature on this topic, we found that vimentin is degraded in the initial phase of tissue damage because of proteolytic activity [32,33,34]. This could explain why our 0 h PTI brain samples were mostly negative for vimentin. To evaluate the temporal expression of the two markers, we also used the Spearman rank correlation test. The analysis showed that the vimentin-immunoreaction has a strong positive correlation with PTI (rho value 0.90, *p*-value < 0.01); in our sample, the longer the PTI, the higher the intensity of vimentin positivity. On the other hand, p53-immunoreactivity did not show a correlation with the PTI (rho value 0.0987, *p*-value > 0.05). The correlation between the two variables was very weak and p53-immunoreaction varied slightly by duration, but this tendency was not supported by a sufficient significance level (*p*-value > 0.05).

We are conscious of the limitations of the present study. In primis, the great limitation of our research is represented by the small sample size. We collected only 12 TBI cases, which is not enough to provide strong scientific evidence. We only selected 12 cases from the autopsy database because the majority of TBI cases were autopsied more than 36 h after death due to reasons related to the time requested by the judicial proceedings. The inclusion of such cases would lead to bias related to the initial putrefaction of the body. In the initial phase of the research in this field, which has not been investigated before, we think it is fundamental to find objective diagnostic criteria and markers not influenced by variables other than TBI and PTI. In future research, the evaluation of diagnostic markers of TBI in different conservative states/putrefactive phases of the bodies would need to be performed. Another limitation of the study we conducted is the possible influence of comorbidities of the subjects. Indeed, as previously described, vimentin expression is demonstrated to be enhanced in some degenerative brain diseases [31]. Since this is a retrospective study using an already-produced database, it is not possible to recall information that was not previously collected. So, it was not possible to evaluate the presence of comorbidities in all the cases. A hypothesis that should be further investigated is whether vimentin is still reliable when the age of the subject is advanced, for example, over 80 years old. Despite these great limitations, the present study is one of the few research projects on this topic. The identification of a pool of markers to confirm the diagnosis of TBI is fundamental in forensic pathology for several reasons. The first is that when a criminal prosecution occurs, the reconstruction of the events and death modality should be based on strong evidence. Therefore, the use of immunohistochemical analysis to scientifically prove the diagnosis of TBI, and eventually the timing of the trauma, could be a great support. Indeed, the use of non-truculent images in the court could be an advantage for the effectiveness of the medico-legal dissertation. The identification of post-mortem markers of TBI may provide support for clinical studies in the evaluation of the same markers for clinical, diagnostic, and therapeutic purposes. For example, better insight into the molecular pathways that induce vimentin and p53 overexpression in TBI cases may be useful in the identification of new pharmacological targets to slow down the progression of brain damage. Furthermore, the potential correlation between vimentin and PTI obtained by our study is the first step in the identification of a pool of markers with time-dependent expression. This means they may be used as a “biological clock” aimed at dating head trauma.

## 5. Conclusions

The vitality evaluation and timing estimation of TBI still represent an issue for forensic pathologists. When the time interval between trauma and death is short, verifying the presence of vital reactions can be difficult. Certainly, the study presented in this paper needs to be contextualized in actual scientific scenarios as not many molecules and markers have been tested for this purpose. The present research has the limitation of a small sample size; however, the tested molecules (vimentin and p53) have shown great potential for use as biological markers, in addition to others, for the diagnosis and timing of TBI.

Recent novelties in this field highlight the necessity of adopting correct and personalized methodological approaches in cases of vitality diagnosis and timing evaluation of lesions. Vimentin and p53, according to our results, represent possible markers that need to be further investigated. The identification of a panel of antibodies that could be used in vitality and timing estimation in cases of TBI is promising in providing solid scientific evidence for the diagnosis of TBI and proving, beyond a reasonable doubt, the timing of trauma.

## Figures and Tables

**Table 1:** Main characteristics of the subjects included in the study group. PTI, post-trauma interval; PMI, post-mortem interval.

Case	Age/Sex	Traumatic Event	PTI/PMI	Autopsy Findings
1	56/m	Neck and head trauma after assault	4 days/24 h	Brain contusion, subarachnoid hemorrhage, and carotid dissection
2	16/m	Traffic accident with traumatic brain injury	0 h/26 h	Brain contusion and laceration, subarachnoid hemorrhage, edema, head fracture, leg fracture and various skin wounds
3	71/f	Traffic accident	12 days/30 h	Brain contusion and hypoxic-ischemic brain injury following cardiac arrest
4	48/m	Traffic accident with cerebral death	2 days/25 h	Brain contusion
5	49/m	Traffic accident	4 days/25 h	Brain contusion
6	49/m	Fall from height and heroin abuse	0 h/24 h	Brain swelling and pulmonary edema
7	5/m	Fall from height	0 h/24 h	Brain contusion and head fracture
8	47/m	Head assault	0 h/28 h	Brain contusion and laceration, subarachnoid hemorrhage, edema
9	41/m	Head assault	0 h/24 h	Bran contusion and laceration, subarachnoid hemorrhage, edema
10	71/m	Traffic accident car-car	5 days/27 h	Subdural hematoma, bran contusions, subarachnoid hemorrhage and secondary brain injury
11	29/m	Fall from height with alcohol intoxication	0 h/36 h	Brain swelling and contusion, thorax fracture, heart rupture
12	80/f	Traffic accident car-car	0 h/25 h	Brain contusion, subdural hemorrhage, hands and foot fracture, sternal bruises

**Table 2:** Description of the gradation used for the semi-quantitative analysis.

Grade	Meaning
0	Absence of staining
+1	Minimal staining in minor areas/spots
+2	Clear staining in more diffuse areas
+3	Diffuse intense staining

**Table 3:** The results of the immunohistochemical analysis of the 12 cases selected in this study.

Cases	Age	PTI	Vimentin	p53
1	56 y.o.	4 days	Positive astrocytes near the vessels	Positive neurons near the hemorrhagic areas
2	16 y.o.	0 h	Negative	Negative
3	71 y.o.	12 days	Positive astrocytes within the cortex	Positive spots within the neurons
4	48 y.o.	2 days	Positive astrocytes near the vessels	Positive neurons near the hemorrhagic areas
5	49 y.o.	4 days	Positive astrocytes near the vessels	Positive spots within the neurons
6	49 y.o.	0 h	Negative	Negative
7	5 y.o.	0 h	Negative	Positive spots within the neurons
8	47 y.o.	0 h	Negative	Positive neurons near the hemorrhagic areas
9	41 y.o.	0 h	Negative	Positive neurons near the hemorrhagic areas
10	71 y.o.	5 days	Positive astrocytes near the vessels	Positive spots within the neurons
11	29 y.o.	0 h	Negative	Negative
12	80 y.o.	0 h	Positive astrocytes near the vessels	Positive neurons near the hemorrhagic areas

**Table 4:** The results of the immunohistochemical analysis of the 12 cases selected in this study applying the semi-quantitative scale shown in the materials and methods section.

Cases	Age	PTI	Vimentin Grade	p53 Grade
1	56 y.o.	4 days	+1	+3
2	16 y.o.	0 h	0	0
3	71 y.o.	12 days	+2	+2
4	48 y.o.	2 days	+1	+ 3
5	49 y.o.	4 days	+2	+1
6	49 y.o.	0 h	0	0
7	5 y.o.	0 h	0	+2
8	47 y.o.	0 h	0	+3
9	41 y.o.	0 h	0	+3
10	71 y.o.	5 days	+2	+2
11	29 y.o.	0 h	0	0
12	80 y.o.	0 h	+1	+3

**Table 5:** The results of the immunohistochemical analysis of the 12 deceased included in the control group.

Controls	Vimentin Grade	p53 Grade
13	0	0
14	0	0
15	0	0
16	0	+1
17	0	0
18	0	0
19	0	0
20	0	0
21	0	0
22	0	0
23	0	0
24	+1	0

**Table 6:** Contingency table of the two variables (vimentin and p53 immunoreaction) among the two groups (TBI cases and controls). TBI, traumatic brain injury.

	Vimentin Positive	Vimentin Negative	p53 Positive	p53 Negative	Total
TBI group	6	6	9	3	12
Control group	1	11	1	11	12
Total	7	17	10	14	24

## Data Availability

The original contributions presented in this study are included in the article. Further inquiries can be directed to the corresponding author.
